# Does a combined intravenous-volatile anesthesia offer advantages compared to an intravenous or volatile anesthesia alone: a systematic review and meta-analysis

**DOI:** 10.1186/s12871-021-01273-1

**Published:** 2021-02-15

**Authors:** Alexander Wolf, Helene Selpien, Helge Haberl, Matthias Unterberg

**Affiliations:** 1 Ruhr University Bochum, Bochum, Germany; 2 Department of Anesthesiology, Intensive Care and Pain Medicine, Universitätsklinikum Knappschaftskrankenhaus Bochum GmbH, In der Schornau 23 – 25, 44892 Bochum, Germany

**Keywords:** Combined intravenous volatile anesthesia, CIVA, Meta-analysis, General anesthesia, PONV, Postoperative pain, Time to extubation

## Abstract

**Background:**

In anesthesia, additive drug interactions are used for reducing dose and dose-dependent side-effects. The combination of propofol with volatile anesthetics is rather unusual but might have advantages compared to the single use regarding PONV, time to extubation, movement during surgery and postoperative pain perception.

**Methods:**

We searched PubMed, Scopus, Web of Science, and CENTRAL for relevant studies comparing combined intravenous volatile anesthesia with total intravenous or balanced anesthesia. The studies identified were summarized in a meta-analysis with the standardized mean difference or risk ratio as the effect size.

**Results:**

Ten studies provided data. The risk for PONV in the recovery room was significantly reduced for a combined anesthesia compared to a balanced anesthesia (RR 0.657, CI 0.502–0.860, *p*-value 0.002). There was no significant difference detected either in the time to extubation or in pain perception. Movement during surgery was significantly reduced for a combined compared to a total intravenous anesthesia (RR 0.241, CI 0.135–0.428, *p*-value 0.000).

**Conclusions:**

The combination of propofol and volatiles may have some advantages in the early occurrence of PONV compared to a balanced anesthesia. To sufficiently evaluate potential advantages of a combination of volatiles and propofol further high-quality trials are needed.

**Trial registration:**

PROSPERO CRD42019126627.

**Supplementary Information:**

The online version contains supplementary material available at 10.1186/s12871-021-01273-1.

## Introduction

Combinations of different drugs, acting synergistically or in addition to one another, are commonly used in anesthesia: opioids in combination with hypnotics. Recent strategies of anesthesia mainly use these synergistic drug-interactions to reduce the dose and dose dependent side-effect of single substances. Another example are benzodiazepines used as premedication with additive effects on hypnosis induction and maintenance. The combination of volatile anesthetics like iso-, sevo- or desflurane with propofol is less common and maybe underestimated in its benefit although these two drugs work additively and have different elimination pathways. These properties might be beneficial compared to the use of one agent alone. In the following meta-analysis we compared the combination of intravenous and volatile anesthetics (CIVA) with a total intravenous anesthesia (TIVA) and a balanced anesthesia (BAL) regarding the occurrence of PONV, time to extubation, movement during surgery and pain perception.

## Methods

The study protocol of this meta-analysis was registered at PROSPERO (International prospective register of systematic reviews; https://www.crd.york.ac.uk/prospero/; registration number CRD42019126627).

We searched for trials without any restriction in the databases PubMed, Scopus, Web of Science, and CENTRAL. We used the search terms “sevoflurane AND propofol”, “desflurane AND propofol”, “isoflurane AND propofol”, “volatile AND propofol”, “inhal AND propofol”, “combined intravenous volatile”, and “CIVA”. Additionally, references of relevant studies were screened as well as current literature.

Only controlled studies, investigating the effect of combined intravenous volatile anesthesia (CIVA) versus balanced (BAL) or total intravenous anesthesia (TIVA) in English or German language and providing data on postoperative nausea and vomiting (PONV), time to extubation, or pain perception were included.

If a study had more than one active drug arm, data were extracted for each treatment arm and included separately in the analysis. Duplicate use of the same placebo group was then automatically factored in by the meta-analysis software used.

Furthermore, randomized and non-randomized studies were analyzed and compared separately.

All complete papers reporting trials were rated independently by two investigators (M.U. and A.W.). Data were extracted onto standard simple forms. Any disagreement was discussed with additional reviewers (HS, HH), and decisions were documented. If necessary, authors of studies were contacted for clarification. The risk of bias was assessed on a sectoral basis: generation of random sequences, concealment of assignments, blinding, incomplete result data, selective reporting.

The primary outcome was PONV in the post anesthesia care unit (PACU) or recovery room (RR). The secondary outcome was PONV within 24 h, time to extubation, movement during surgery, pain intensity in the PACU/RR and pain intensity within 24 h.

### Statistical analysis

We analyzed pooled studies using BAL and pooled studies using TIVA, as well as the overall effect. In a sensitivity analysis we excluded non-randomized studies and considered only randomized controlled trials.

The outcome data were combined in a meta-analysis. We calculated the risk ratio (RR) for dichotomous data such as the occurrence of PONV and movement during surgery. For continuous data like time to extubation and pain intensity we calculated the standardized mean difference (SMD) and their 95% confidence interval (CI) as effect size measure.

We used the model of random effects due to the inhomogeneity of the studies themselves, such as different types of surgery (thoracic, laparoscopic, ear/nose/throat) and study populations (gynecological vs. non-gynecological) and due to different heterogeneous results in the studies. Study heterogeneity was assessed by a Chi-square test and the I-square statistic [1]. The Chi-square test compares the effect sizes of the individual trials with the pooled effect size. Significance levels of *p* < 0.1 were determined a priori in order to assume the presence of heterogeneity. The I-square statistic provides an estimate of the percentage of variability due to heterogeneity rather than chance alone. We interpreted values ≥50% as considerable heterogeneity [1]. If the results were statistically significantly heterogeneous, reasons for the heterogeneity were searched for by re-reading the publications, verifying the extracted data and looking for deviations in the study methodology that explain the heterogeneity. Small studies with negative results are less likely to be published than studies with significant results. The possibility of such a publication bias was examined using the funnel plot method described by Egger et al. [2].

The meta-analytical calculations were performed using the Comprehensive Meta-analysis version 3. The exact formulas are reported there [3]. A *p*-value < 0.05 was considered statistically significant.

## Results

### Search strategy

**Fig. 1:**
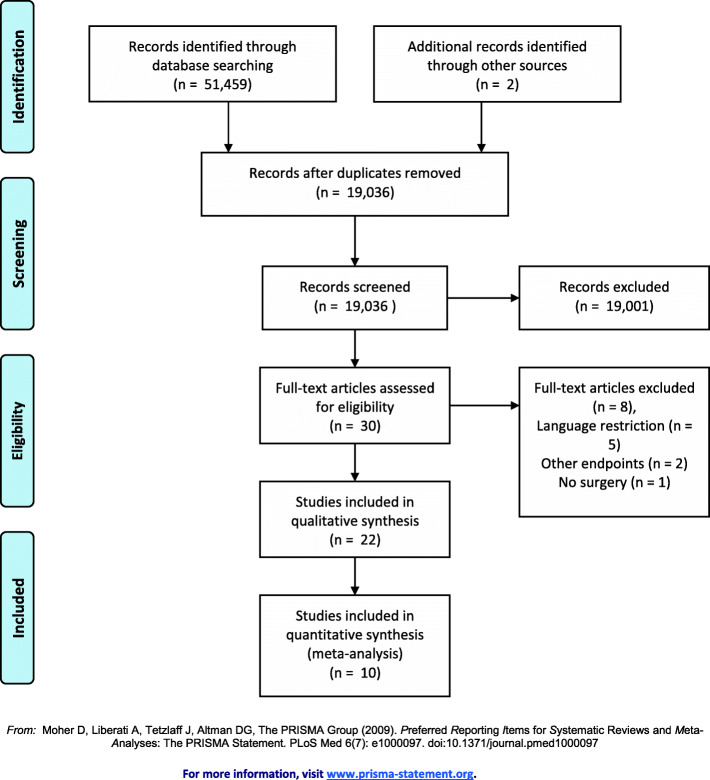
PRISMA flow diagram displaying the search and extraction process

We screened 19,036 records, of which 30 were intensively evaluated (see Fig. 1). Ten studies provided data on the occurrence of PONV in the PACU/RR, PONV within 24 h, time to extubation, movement during surgery, pain intensity in the PACU/RR and pain intensity within 24 h. All included studies provided data on PONV [4–13], five on time to extubation [5, 6, 8, 9, 13], two on movement during surgery [6, 8] and four on postoperative pain [4, 6, 7, 10]. Liang et al. [9] and Chen et al. [4] presented their data on pain only categorized, whereas Hensel et al. provided data only as median with confidence interval [6]. The corresponding authors of these studies were contacted via the email address given in the publication and were kindly asked to provide us with the mean values and standard deviations. The only author who responded was Dr. Hensel [6] whom we thank.

### Included studies and participants

Ten studies with 16 treatment arms and 1960 participants were included. These studies reached a mean value of 5.7 points (standard deviation 1.1) and a median of 6 points (range 3–7) in the Delphi list for quality assessment [14]. For a detailed overview, see supplementary Table 1. A detailed overview of the included studies is given in Table 1.

**Table 1:** Overview of study characteristics

First author, Year, Origin	Surgery	ASA	Premedication/ antiemetics/ regional anesthesia	CIVA	TIVA	BAL	EEG Target
Chen 2016 Taiwan	Laparoscopic cholecystectomy	I – II	–	**Induction:** 2 μg/kg fentanyl, 1 mg/kg lidocaine, 2 mg/kg propofol, 0,8 mg/kg rocuronium **Maintainance:** 3% et desflurane, propofol TCI (Ce 4 μg/ml), 1 μg/kg/h fentanyl	**Induction:** 2 μg/kg fentanyl, 1 mg/kg lidocaine, 2 mg/kg propofol, 0,8 mg/kg rocuronium **Maintainance:** Propofol TCI (Ce 4 μg/ml), 1 μg/kg/h fentanyl	–	–
Chi 2012 China	Laparoscopic gynecologic	I – II	0,01 mg/kg atropine 2 mg/kg luminal	**Induction:** 4 μg/kg fentanyl, 0,05 mg/kg midazolam, 1,5 mg/kg Propofol, 0,1 mg/kg vecuronium **Maintainance:** 0,6–1 MAC isoflurane, 2–3 mg/kg/h propofol, 0,1–0,15 μg/kg/min remifentanil	**Induction:** 1 μg/kg remifentanil, 0,05 mg/kg midazolam, 1,5 mg/kg propofol, 0,1 mg/kg vecuronium **Maintainance:** 5–10 mg/kg/h propofol, 0,2–0,3 μg/kg/min remifentanil	**Induction:** 4 μg/kg fentanyl, 0,05 mg/kg midazolam, 1,5 mg/kg Propofol, 0,1 mg/kg vecuronium **Maintainance:** 1–2,5 MAC isoflurane, 1 μg/kg fentanyl per hour	BIS 40–60
Hensel 2019 Germany	Elective surgery	I – III	0,05–0,15 mg/kg midazolam Apfel-Score 2–3: 4 mg dexamethason Apfel-Score 4: 4 mg dexamethason plus 4 mg ondansetrone	**Induction:** 2–3 mg/kg Propofol, 0,3–0,5 mg rocuronium **Maintainance:** 1–3% et sevoflurane, propofol TCI Snider Ce 1 μg/ml, 0,15–0,3 μg/kg/min remifentanil	**Induction:** 2–3 mg/kg Propofol, 0,3–0,5 mg rocuronium **Maintainance:** Propofol TCI Snider Ce 2–4 μg/ml, 0,15–0,3 μg/kg/min remifentanil	–	Narcotrend 30–60
Kawano 2016 Japan	Laparoscopic gynecologic	I – II	–	**Induction:** remifentanil, propofol, rocuronium **Maintainance:** 0,5 MAC sevoflurane, 2 mg/kg/h propofol, remifentanil	**Induction:** remifentanil, propofol, rocuronium **Maintainance:** 4–8 mg/kg/h propofol, remifentanil	**Induction:** remifentanil, thiamylal, rocuronium **Maintainance:** 1 MAC sevoflurane, remifentanil	BIS 40–60
Lai 2017 Taiwan	Ocular surgery	I - III	5 mg dexamehasone	**Induction:** propofol TCI Schneider Ce 4 μg/ml, 2 μg/kg fentanyl, 1.5 mg/kg lidocaine **Maintainance:** 1% inspiratory sevoflurane, propofol TCI Schneider	**Induction:** propofol TCI Schneider Ce 4 μg/ml, 2 μg/kg fentanyl, 1.5 mg/kg lidocaine **Maintainance:** propofol TCI Schneider		BIS 40–60
Lai 2018 Taiwan	Video-assisted thoracoscopic surgery	I - III	Thoracic epidural catheter	**Induction:** propofol TCI Schneider Ce 4 μg/ml, 0,1 mg fentanyl **Maintainance:** 1% inspiratory sevoflurane, propofol TCI Schnider Ce 4 μg/ml	**Induction:** propofol TCI Schneider Ce 4 μg/ml, 0,1 mg fentanyl **Maintainance:**: propofol TCI Schnider Ce 4 μg/ml	–	BIS 40–60
Liang 2014 China	Gastro-intestinal surgery	I – II	Epidural catheter	**Induction:** propofol TCI Schnider Cp 4 μg/ml, remifentanil 0,15 μg/kg/min, fentanyl 1 μg/kg, rocuronium 0,6–0,8 mg/kg **Maintainance:** 0,3 MAC sevoflurane, propofol TCI Schnider Cp 1,2 μg/ml, intermittent 50 μg fentanyl and 10 mg rocuronium	**–**	**Induction:** propofol TCI Schnider Cp 4 μg/ml, remifentanil 0,15 μg/kg/min, fentanyl 1 μg/kg, rocuronium 0,6–0,8 mg/kg **Maintainance:** sevoflurane, 50 μg intermittent fentanyl and 10 mg rocuronium	BIS 40–60
Van den Berg 1995 Saudi Arabia	Tympanoplasty, septorhinoplasty, adenotonsillectomy	I – II	0,3 mg/kg diazepam (maximum 20 mg), 0,15 mg/kg metoclopramide (maximum 10 mg)	**Induction:** 2 mg/kg propofol, 0,5 mg/kg lignocaine 2%, 0,6 mg/kg atracurium, 0,2 mg/kg nalbuphine **Maintainance:** 0,6–0,8% insp. Isoflurane, 10 mg/kg/h propofol, after 15 min reduction to 5 mg/kg/h propofol	**Induction:** 2 mg/kg propofol, 0,5 mg/kg lignocaine 2%, 0,6 mg/kg atracurium, 0,2 mg/kg nalbuphine **Maintainance:** 10 mg/kg/h propofol, after 15 min reduction to 5 mg/kg/h propofol	**Induction:** 4,0 mg/kg thiopentone, 0,5 mg/kg lignocaine 2%, 0,6 mg/kg atracurium, 0,2 mg/kg nalbuphine **Maintainance:**: 0,6–0,8% insp. Isoflurane	-
Won 2011 Republic of Korea	Thyreodectomy	I – II	–	**Induction:** 1% sevoflurane, propofol TCI Cp 1,5–2,5 μg/ml, remifentanil TCI Cp 2,5–3,5 ng/ml, 0,6 mg/kg rocuronium **Maintainance:** 1% sevoflurane, propofol TCI Cp 1,5–2,5 μg/ml, remifentanil TCI Cp 2,5–3,5 ng/ml	**Induction:** propofol TCI Cp 2,5–3,5 μg/ml, remifentanil TCI Cp 3,5–4,5 ng/ml, 0,6 mg/kg rocuronium **Maintainance:** propofol TCI Cp 2,5–3,5 μg/ml, remifentanil TCI Cp 3,5–4,5 ng/ml	**Induction:** 4–5 mg/kg thiopental, 0,6 mg/kg rocuronium **Maintainance:** 2–2,5% sevoflurane	BIS
Zhang 2013 China	Laparoscopic gynecologic	I – II	0,1 g phenobarbital i.m. 0,5 mg atropin i.m. Seperate groups (CIVA/TIVA/BAL) each with ondansetron 8 mg or placebo	**Induction:** 2 mg/kg prpofol, 5 μg/kg fentanyl, 0,1 mg/kg vecuronium **Maintainance:** sevoflurane, propofol	**Induction:** 2 mg/kg prpofol, 5 μg/kg fentanyl, 0,1 mg/kg vecuronium **Maintainance:** 10 mg/kg/h propofol	**Induction:** 2 mg/kg prpofol, 5 μg/kg fentanyl, 0,1 mg/kg vecuronium **Maintainance:** 2–3% sevoflurane	-

*et* end tidal, *i.m.* intra muscular, *BIS* Bispectral index, *TCI* target controlled infusion, *Ce* effect site concentration, *Cp* plasma concentration, *MAC* minimal alveolar concentration

### Outcome

Four studies with six treatment arms [4, 7, 9, 11] provided data on **PONV in the PACU/RR** (three arms each CIVA vs. BAL and CIVA vs. TIVA). The overall risk for PONV in the PACU/RR was significantly reduced for the CIVA group (RR 0.657, CI 0.502–0.860, *p*-value 0.002), compared to the BAL group. Thus, CIVA showed a significant risk reduction for PONV in the PACU/RR (RR 0.514, CI 0.364–0.725, *p*-value 0.000). Comparing CIVA to TIVA no difference between the groups (RR 0.970, CI 0.629–1.497, *p*-value 0.892) was found. There was no heterogeneity (Q-value 6.74, df (Q) 5, *p-*value 0.24, I^2^ 25.86).

**Fig. 2:**
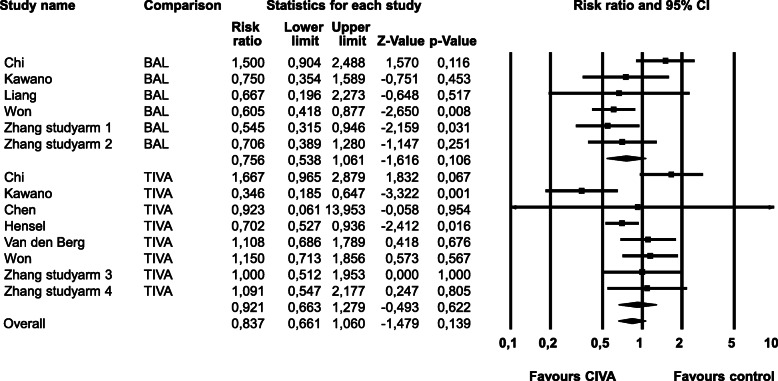
PONV within 24 h

The risk for **PONV within 24 h** postoperatively is shown in Fig. 2. A significant heterogeneity (Q-value 29.86, df (Q) 13, *p*-value 0.00, I^2^ 56.47) was found. In a sensitivity analysis we removed the only non-randomized study [6] which had only TIVA as control. The subgroup compared to TIVA showed no significant change (RR 0.980, CI 0667–1.440, *p*-value 0.916).

**Fig. 3:**
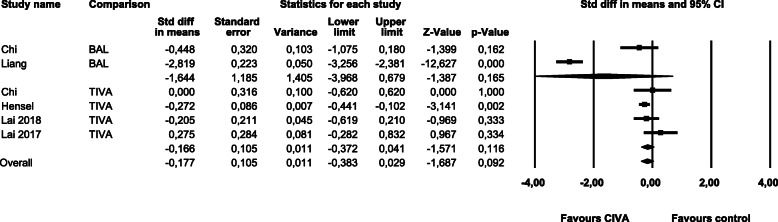
Time to extubation

Results on **time to extubation** are shown in Fig. 3. Here we found a significant heterogeneity (Q-value 126.63, df (Q) 5, *p-*value 0.00, I^2^ 96.05). In a sensitivity analysis the non-randomized study was removed, but the TIVA subgroup (SMD -0.026, CI -0.319 – 0.267, *p*-value 0.860) nor the overall results (SMD -0.052, CI -0.342 – 0.239, *p*-value 0.727) were significantly altered.

Only two studies provided data on **movement** during surgery [6, 8]. Both studies used TIVA as a control group and both studies point in the same direction leading to a significant overall result in favor of CIVA (RR 0.241, CI 0.135–0.428, *p*-value 0.000). No heterogeneity was found (Q-value 0.31, df (Q) 1, *p*-value 0.57, I^2^ 0.00). Since only two studies were included in this analysis, we have omitted the sensitivity analysis.

Two studies with three treatment arms provided data on **pain in the PACU/RR** [6, 7]. There was neither a significant difference between CIVA and balanced anesthesia (SMD -0.181, CI -0.610 – 0.248, *p*-value 0.408) nor between CIVA and TIVA (RR 0.071, CI -0.086 – 0.228, *p*-value 0.376). The results of the subgroup reflect the overall effect without significant difference (SMD 0.041, CI -0.106 – 0.188, *p*-value 0.585). We found no heterogeneity (Q-value 1.21, df (Q) 2, *p-*value 0.55, I^2^ 0.00). The removal of the non-randomized study did not significantly alter the overall effect (SMD -0.034, CI -0.337 – 0.269, *p*-value 0.825).

**Fig. 4:**
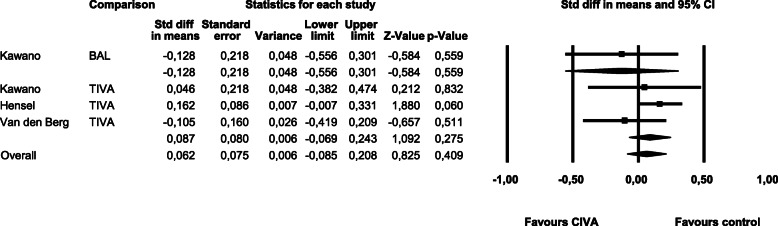
Pain within 24 h post surgery

The results for **pain** in a period of **24 h** after surgery are shown in Fig. 4. We found no heterogeneity (Q-value 3.16, df (Q) 3, *p-*value 0.37, I^2^ 5.17). The removal of the only non-randomized study had no significant impact on the overall results (SMD -0.072, CI -0.290 – 0.146, *p-*value 0.519).

## Discussion

The combination of two different hypnotics, namely volatile anesthetics and propofol to maintain anesthesia, is rather unusual. Nevertheless, from a pharmacological and practical point of view the combination of these two agents might be useful.

Even in subhypnotic doses propofol is known to have antiemetic properties [15]. Further there is a degree of exposure dependent effect of volatiles on PONV occurrence [16]. However, this may explain the significantly lower PONV rates in patients with CIVA compared to balanced anesthesia in the RR. An analysis comparing CIVA with BAL within 24 h shows only a non-significant result. Interestingly, CIVA is comparable to TIVA in regards of risk for PONV. These results have to be interpreted cautiously, as a significant heterogeneity was evident. Apart from statistical heterogeneity there are variable factors which may influence the occurrence of PONV due to non-standardized anesthetic practice. The choice of opioid for anesthesia induction, maintenance and postoperative pain therapy might have influenced the occurrence of PONV [17, 18]. Some of the included studies applied different opioids for the different treatment groups like fentanyl and remifentanil for anesthesia induction and maintenance [5]. The postoperative pain therapy strategy varied in the choice of substances and was inconsistently reported. Another factor to consider was the induction agent used for anesthesia. Two studies used barbiturates in the BAL group [7, 11] with a greater likelihood of PONV [19, 20] and a possible overestimation of the PONV reducing effect of CIVA compared to BAL. However, the risk for postoperative vomiting due to volatiles is restricted to the early postoperative hours [16] suggesting a PONV preventive effect by adding propofol to volatiles in the early postoperative period. This PONV preventing effect diminishes within 24 h. It remains unclear if this is due to the propofol clearance under a certain plasma level threshold and prolonged effect of volatiles on the area postrema, or if there are other factors influencing the occurrence of PONV. Not all included studies reported on established risk-factors of PONV like e.g., smoking or proportion of female patients, so that there might be a disbalance between the groups especially in those studies with small group sizes although a randomization has been performed.

Propofol and volatile anesthetics such as sevoflurane act additively [21, 22], and the primary organ of elimination for propofol is the liver, whereas volatiles are eliminated through the lungs. Theoretically the use of lower doses of two additive hypnotics with different elimination pathways should result in a shorter postanesthetic recovery time, which is reflected by the time to extubation. In this meta-analysis we found no difference between the combination of intravenous and volatiles anesthetics. However, some studies (Liang and Hensel) indicate a positive effect for CIVA. The overall effect might be diminished due to the fact that all included studies used a processed intraoperative electroencephalogram to measure the depth of hypnosis. In addition, Propofol TCI was frequently used, resulting in a very precise control of hypnosis depth and regain of consciousness in the TIVA group. This could be one reason why there is only a small positive effect evident for the CIVA regime. Thus, the advantage could be greater compared to anesthesia without measuring the depth of hypnosis and with a conventional propofol infusion pump. It is striking, however, that the study with greatest benefit for the CIVA group focused on patients undergoing major abdominal operations, namely intestinal and gastric surgery, which result in a longer duration of anesthesia. The average duration of surgery was approximately 60–70 min longer than in the study by Chi and Hensel et al. and 90 min longer than in the study of Lai et al. Thus, if only one hypnotic drug is used, a prolonged surgery or anesthesia can lead to a higher accumulation in the body, resulting in a longer elimination time. Here an advantage for combined intravenous volatile anesthesia could therefore arise. To prove this, we performed a meta-regression, which showed a strong correlation between duration of surgery and time to extubation with a *R*^2^ = 0.89 (*p* = 0.000) (see supplementary Fig. 1). The combination of two hypnotics could have a positive effect on postanesthetic recovery time and time to extubation depending on the duration of anesthesia. However, there are inconsistent conditions about termination of the administration of anesthetics among the included studies. Chi et al. did not state the conditions of termination [5]. Hensel et al. defined the time to extubation as the time point from which the anesthetic administration was completely terminated [6]. We assume that the administered amount of sevoflurane and/or propofol may have been reduced when the end of surgery has been anticipated. Liang et al. defined the starting timepoint as the turn-off of anesthetics administration after surgery was complete [9]. Extubation was performed with a BIS value above 70 and spontaneous breathing. Lai et al. stopped anesthetic administration at the end of procedure and extubated after consciousness was regained [8, 13]. These unequal conditions restrict the findings in the regression analysis.

When comparing CIVA to TIVA, we found less movement during surgery in the CIVA group. Volatile anesthetics act inter alia on the spinal cord and suppress movement [23]. This effect is significantly more pronounced for volatiles than for propofol [24, 25], which may lead to a more favorable outcome when sevoflurane or isoflurane is added to propofol. Movement during surgery might further depend on muscle relaxation and intraoperative pain control. Only two studies delivered data on movement during surgery. The study by Hensel and colleagues included 270 patients per group in various surgical procedures. They only used 0.3–0.5 mg of rocuronium once with anesthesia induction and they reported no significant difference for the intraoperative remifentanil consumption between the CIVA and TIVA group. But they observed movements during surgery in 3% vs. 14% (CIVA vs. TIVA) of the patients. The study by Lai and colleagues investigated CIVA vs. TIVA in non-intubated video-assisted thoracoscopic surgery (VATS) [8]. They used laryngeal mask airway while muscle relaxants were not used. For pain management all patients received a thoracic epidural anesthesia with additional surgical intercostal blocks. The TIVA group showed a significantly higher intraoperative fentanyl consumption than the CIVA group (145 vs. 128 μg). The patients with TIVA had significantly higher rates of movement compared to the CIVA group (17 vs. 5). This limited data suggests a possible benefit for adding volatiles to suppress movements during surgery. However, more high-quality studies are needed to draw further conclusions.

Postoperative pain differed neither between CIVA and BAL nor between CIVA and TIVA. However, some studies, which investigated postoperative pain comparing TIVA to BAL, showed a beneficial effect on postoperative pain and opioid intake in TIVA. A meta-analysis by Peng and colleagues addressed this topic and found a statistically significant benefit for propofol with questionable clinical relevance. This result was accompanied by a significant heterogeneity [26]. A recent study investigating the effect of propofol on post-sternotomy pain found no effect on acute or chronic pain [27]. Postoperative pain perception is more likely to be influenced by the use of a multi-modal pain management. Dexamethasone has a strong anti-inflammatory potential and is a useful co-analgetic [28]. The purpose for using dexamethasone in the included studies was PONV prevention. Only two studies provided data on postoperative pain [6, 7] of which only one used dexamethasone risk stratified in according to the PONV risk [6]. The use of dexamethason might have influenced postoperative pain perception, but as there was no significant difference in PONV risk score between the groups, the effect of dexamethasone should be equally adjusted. Barbiturates have been associated with hyperalgesia [29]. The study by Kawano et al. used the barbiturate thiamylal for anesthesia induction only for the BAL group. Recent research could not find evidence supporting the association between barbiturates and hyperalgesia [30]. So, an influence of barbiturates on measured pain is rather unlikely.

The strength of this study is to be the first meta-analysis to address this topic. We included 10 studies with 1960 patients. However, the studies included are of moderate to low quality with significant heterogeneity, which limits the significance of our results. Apart from statistical heterogeneity there is also a relevant heterogeneity from a clinical point of view. Besides the different surgical interventions, there is a huge variability in anesthetic management between the studies. Solely a small number of studies used premedication, PONV risk was inconsequently reported and PONV prophylaxis was carried out by some, while others prescribed dexamethasone to all patients. All TIVA and CIVA patients received propofol for induction of anesthesia. The BAL patients received among propofol also barbiturates. The intraoperative analgesia concepts contained lidocaine, fentanyl, remifentanil, nalbuphine and regional anesthesia and the procedure at the end of surgery with regard to turning off the anesthetic agents differed between the studies. The lack of standardization limits the comparability and explanatory power of the CIVA concept.

However, an anesthetic regimen with a comparable PONV incidence to TIVA, with less intraoperative movements and with a shorter time to extubation would be desirable from a patient, surgical and economic view. Therefore, we suggest a thoroughly planned multi-center randomized controlled trial to compare the different concepts. This study should include three treatment arms: CIVA, TIVA and BAL for standardized surgical procedures. Also a standardized anesthetic concept (including standardized risk adapted PONV prophylaxis and standardized pain control) using a processed electroencephalogram with a predefined anesthesia depth and remifentanil as sole opioid should be implemented. The CIVA concept uses two different anesthetics to reduce the overall dose and dose dependent side-effect of single substances use. Nevertheless, side effects could be relevant and should be monitored as well as its cost effectiveness.

## Conclusions

CIVA showed a similar risk for PONV in the recovery room compared to a TIVA and in the early postoperative period a reduced risk compared to a BAL. However, this effect was not consistent for the first 24 postoperative hours with no difference between CIVA and BAL. The CIVA showed lower rates of intraoperative movements compared to a TIVA with the major limitation of only two studies providing data. These results must be seen in the context of moderate to low study quality with significant heterogeneity. We suggest carrying out a sufficiently powered multi-center randomized controlled trial to evaluate reasonable benefits for a combination of propofol and volatile anesthetics.

## Supplementary Information

**Additional file 1: Supplemental Fig. 1**: Meta-regression correlating time to extubation and surgery duration with bold correlation line, confidence interval (slim lines) and individual studies (circles)

**Additional file 2: Supplemental Table 1**: Delphi List for Quality Assessment of Randomized Clinical Trials

## Data Availability

The datasets used and analysed during the current study available from the corresponding author on reasonable request.

## Abbreviations

CIVA: Combined intravenous volatile anesthesia
TIVA: Total intravenous anesthesia
BAL: Balanced anesthesia
RR: Risk ratio
SMD: Standardized mean difference
CI: Confidence interval
PONV: Postoperative nausea and vomiting
PACU: Post anesthesia care unit
RR: Recovery room
Et: End tidal
MAC: Minimal alveolar concentration
i.m.: Intra muscular
BIS: Bispectral index
TCI: Target controlled infusion
Ce: Effect site concentration
Cp: Plasma concentration
