# Supplementary figures and images for: Does a combined intravenous-volatile anesthesia offer advantages compared to an intravenous or volatile anesthesia alone: a systematic review and meta-analysis
Source: BMC Anesthesiol. 2021 Feb 15;21:52. doi: 10.1186/s12871-021-01273-1 (PMC7883423; doi:10.1186/s12871-021-01273-1)

## Slide 1
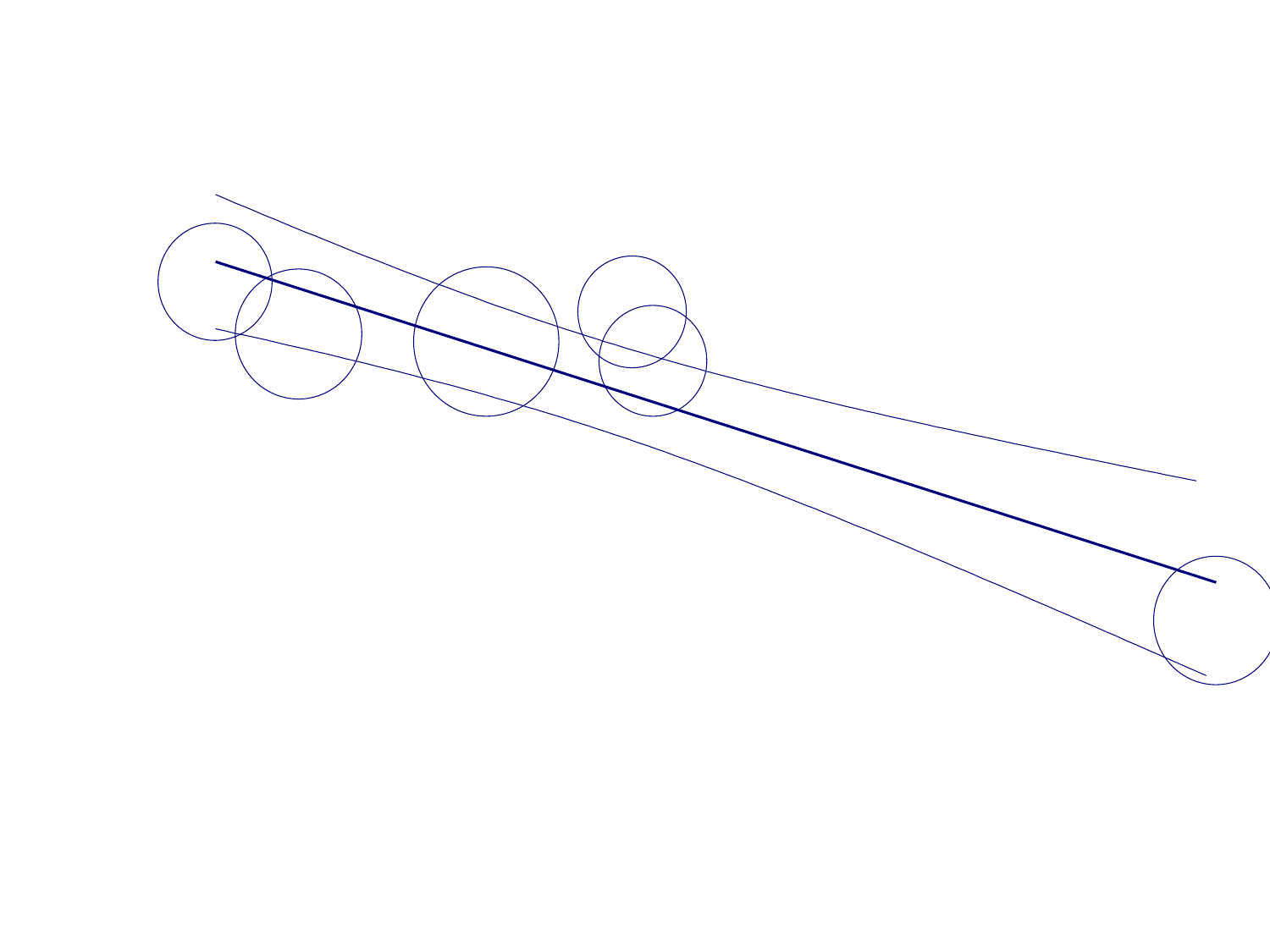

3,00
2,00
1,00
0,00
-1,00
SMD
-2,00
-3,00
-4,00
-5,00
-6,00
40,0
60,0
80,0
100,0
120,0
140,0
160,0
180,0
surgery duration in minutes

Supplement: Supplementary file 1 — Additional file 1: Supplemental Fig. 1: Meta-regression correlating time to extubation and surgery duration with bold correlation line, confidence interval (slim lines) and individual studies (circles) [file 12871_2021_1273_MOESM1_ESM.pptx]
