# Supplementary material for: Does a combined intravenous-volatile anesthesia offer advantages compared to an intravenous or volatile anesthesia alone: a systematic review and meta-analysis
Source: BMC Anesthesiol. 2021 Feb 15;21:52. doi: 10.1186/s12871-021-01273-1 (PMC7883423; doi:10.1186/s12871-021-01273-1)
Supplement: Supplementary file 2 — Additional file 2: Supplemental Table 1: Delphi List for Quality Assessment of Randomized Clinical Trials [file 12871_2021_1273_MOESM2_ESM.docx]

| Criteria | Evaluation | Chen 2016 | Chi 2012 | Hensel 2019 | Kawano 2016 | Lai 2018 | Lai 2017 | Liang 2014 | Van den Berg 1995 | Won 2011 | Zhang 2013 |
| --- | --- | --- | --- | --- | --- | --- | --- | --- | --- | --- | --- |
| 1. Treatment allocation: Was a method of randomization performed? | Yes (1)/No (0) | 1 | 1 | 0 | 1 | 1 | 1 | 1 | 1 | 1 | 1 |
| 2. Treatment allocation: Was the treatment allocation concealed? | Yes (1)/No (0) | 0 | 1 | 0 | 1 | 1 | 1 | 1 | 0 | 1 | 1 |
| 3. Were the groups similar at baseline regarding the most important prognostic indicators? | Yes (1)/No (0) | 1 | 1 | 1 | 1 | 1 | 1 | 1 | 1 | 1 | 1 |
| 4. Were the eligibility criteria specified? | Yes (1)/No (0) | 1 | 1 | 1 | 1 | 1 | 1 | 1 | 1 | 1 | 1 |
| 5. Was the outcome assessor blinded? | Yes (1)/No (0) | 1 | 0 | 0 | 1 | 0 | 0 | 1 | 1 | 1 | 1 |
| 6. Was the care provider blinded? | Yes (1)/No (0) | 0 | 0 | 0 | 0 | 0 | 0 | 0 | 0 | 0 | 0 |
| 7. Was the patient blinded? | Yes (1)/No (0) | 0 | 1 | 0 | 0 | 1 | 1 | 1 | 0 | 0 | 1 |
| 8. Were point estimates and measures of variability presented for the primary outcome measures? | Yes (1)/No (0) | 1 | 1 | 1 | 1 | 1 | 1 | 1 | 1 | 1 | 1 |
| 9. Did the analyses include an intention-to-treat analysis? | Yes (1)/No (0) | 0 | 0 | 0 | 0 | 0 | 0 | 0 | 0 | 0 | 0 |
| Total |  | 5 | 6 | 3 | 6 | 6 | 6 | 7 | 5 | 6 | 7 |
